# Neural Mechanism of Cognitive Reserve in Acupuncture Stimulation: Protocol for a Randomized, Placebo-Controlled Functional Near-Infrared Spectroscopy Trial

**DOI:** 10.2196/66838

**Published:** 2025-02-19

**Authors:** Hyeonsang Shin, Woohyun Seong, Yeonju Woo, Joo-Hee Kim, Kwang-Rak Park, Dong Hyuk Lee

**Affiliations:** 1 College of Korean Medicine Sangji University Wonju-si, Gangwon-do Republic of Korea; 2 Department of Physiology College of Korean Medicine Sangji University Wonju-si, Gangwon-do Republic of Korea; 3 Research Institute of Korean Medicine Sangji University Wonju-si, Gangwon-do Republic of Korea; 4 Department of Acupuncture & Moxibustion College of Korean Medicine Sangji University Wonju-si, Gangwon-do Republic of Korea; 5 Department of Anatomy College of Korean Medicine Sangji University Wonju-si, Gangwon-do Republic of Korea

**Keywords:** cognitive reserve, acupuncture, dementia, mild cognitive impairment, neuroimaging, fNIRS, brain connectivity, neural mechanism, RCT, randomized controlled trial

## Abstract

**Background:**

Dementia is a clinical syndrome characterized by a progressive decline in various cognitive domains. Since there is still no treatment for dementia, early diagnosis and prevention are the best approaches. In this context, the cognitive reserve (CR) concept has received considerable attention in dementia research with regard to prognosis. It originates from discrepancies between the degree of brain pathology and clinical manifestations. Acupuncture, as a complementary intervention, has long been widely applied in neurological diseases in East Asia. At the macroscale level, how acupuncture stimulation affects neural activity concerning CR in normal aging and dementia is largely unknown.

**Objective:**

The aim of this study is to investigate the acute neural mechanisms of acupuncture stimulation concerning CR in the normal aging group and the group with cognitive impairment using neuroimaging methods.

**Methods:**

This study is a randomized, placebo-controlled trial. Participants without (n=30) and with cognitive impairment (n=30) will be randomly assigned to the verum or sham acupuncture groups. The verum acupuncture group will receive acupuncture stimulation at acupoints related to cognitive function and gain deqi sensation. The sham acupuncture group will receive superficial needling at nonacupoints not related to cognitive function. Each group will undergo cognitive function tests, functional near-infrared spectroscopy imaging before and after acupuncture stimulation, and an assessment of CR. The primary outcomes will be differences in resting brain activities according to disease status, differences in resting brain connectivity before and after acupuncture stimulation between the 2 groups, and changes in brain activity in relation to the CR index. The secondary outcomes will be brain connectivity or network metrics associated with CR and differences in neural activity between the cognitive task and resting states.

**Results:**

The recruitment began in August 2023; to date, there have been 50 participants, divided into 20 in the group with cognitive impairment and 30 in the unimpaired group. The recruitment process will continue until February 2025.

**Conclusions:**

CR refers to the individual susceptibility to age-related brain changes and pathologies in cognitive impairment, and it is a factor affecting the trajectories of the disease. Although acupuncture is a widely used intervention for various neurological diseases, including dementia, its mechanism associated with CR at the macroscale has not been clearly identified. This study could contribute to identifying the neural mechanisms of acupuncture stimulation associated with CR using neuroimaging methods and provide a basis for future longitudinal research.

**Trial Registration:**

Clinical Research Information Service of the Republic of Korea KCT0008719; https://tinyurl.com/ydv5537n

**International Registered Report Identifier (IRRID):**

DERR1-10.2196/66838

## Introduction

Cognitive impairment refers to a generic term in which various cognitive functions, such as memory and executive function, are impaired, and the symptom severity ranges from mild to severe enough to interfere with daily life. Among them, dementia is a clinical syndrome preventing patients from leading a daily life due to the severe decline in several cognitive domains. Alzheimer disease (AD) is by far the most frequent cause of dementia and accounts for up to 80% of all dementia diagnoses [1]. AD symptoms typically begin with mild memory difficulties and gradually progress to severe memory impairment, inducing dysfunctions in daily life [2]. The prevalence and incidence of AD are increasing with an increase in life expectancy, leading to a socioeconomic burden worldwide [3,4].

The concept of cognitive reserve (CR) has received consistent attention in dementia-related research. CR refers to the adaptability of cognitive processes, which helps explain the individual vulnerability of cognitive abilities. It has also been suggested as an active model of the reserve, indicating that dynamic cognitive and functional processes counteract aging-related brain changes or damage [5]. This concept suggests that the brain actively attempts to cope with brain damage, allowing an individual with high CR to better deal with brain pathology. Many studies have shown that CR works differently depending on disease status. In the group without cognitive impairment, participants with high CR showed a slower decline in cognitive function; however, in the AD group, individuals with high CR showed more rapid cognitive deterioration than those with low CR [6,7]. Therefore, CR is considered a factor that modulates the relationship between the neuropathological burden of dementia and clinical symptoms [8,9]. Generally, CR has been measured through proxies, such as years of education, occupational complexity, and questionnaires covering various lifelong experiences.

Various neuroimaging techniques such as magnetic resonance imaging, functional magnetic resonance imaging (fMRI), positron emission tomography, and electroencephalography have been used for the early detection of AD [10]. Although fMRI is noninvasive and has good temporal and excellent spatial resolutions among functional neuroimaging methods, it has inherent limitations such as high cost, immobility due to heavy equipment, and vulnerability to head motion artifacts. In contrast, functional near-infrared spectroscopy (fNIRS) has been suggested as an alternative tool for functional neuroimaging. fNIRS is an optical neuroimaging technique that allows the measurement of changes in brain tissue concentrations of oxyhemoglobin (HbO_2_), deoxyhemoglobin (HbR), and total hemoglobin (HbT) within the brain, achieved by irradiating the head with near-infrared (NIR) light [11,12]. This equipment has several practical advantages over conventional techniques represented by fMRI: It has a relatively higher temporal resolution, is noninvasive, cost-effective, lightweight, and easy to handle. It is applicable to cases difficult to fMRI scanning, such as agitated patients, individuals with claustrophobia, and those with pacemakers [13,14]. Moreover, it can be used on patients without moving from the hospital or laboratory, which can be critical for dementia patients with mobility difficulties. With these advantages, it allows us to monitor the alterations in brain function in patients with cognitive impairment in real time and explore neural responses induced by long-term or short-term interventions.

In a recent systematic review of dementia research using fNIRS, its diagnostic and investigative usefulness was evaluated while reviewing 88 studies in the field of dementia [15]. In summary, fNIRS could capture the aberrant hemodynamic responses and a lack of task-appropriate lateralization, often focused on frontal regions, in dementia. On the other hand, inconsistent results were found in prodromal stages. Cognitive decline accompanied by either reduced functional responses or hyperactivity was identified, the latter implying a compensatory response not represented at the dementia stage. However, there have been few fNIRS studies dealing with CR, one of the important factors in cognitive impairment.

Acupuncture has been practiced for thousands of years as an important treatment for a variety of diseases in East Asian medicine [16,17]. It is also being explored in AD research as a complementary and alternative therapy. In a meta-analysis, acupuncture treatment alone showed acceptable efficacy compared to conventional medicine for the management of AD. In addition, in a randomized controlled trial in patients with mild to moderate AD, acupuncture was found to be safe, well tolerated, and effective in improving cognitive function and global clinical status [18]. Specifically, acupuncture has been shown to regulate the release of central neurotransmitters, including acetylcholine and monoamine neurotransmitters [19]. Current neuroimaging studies of acupuncture cover a variety of diseases ranging from pain-related diseases such as chronic low back pain to peripheral and central nervous system disorders including carpal tunnel syndrome, stroke, Parkinson’s disease, and dementia [20-24]. Through these, the analgesic mechanism and neuroprotective effect of acupuncture, which are distinct from sham needling, are consistently elucidated. Among them, a systematic review dealing with 13 AD-related neuroimaging studies found that acupuncture may modulate the default mode network, the central executive, and the frontoparietal networks in the brains of AD patients [24]. However, on the macroscale, the acute neural mechanisms of acupuncture stimulation in relation to CR according to disease status are largely unknown. To identify the neural mechanisms of acupuncture stimulation associated with CR in healthy aging and cognitive impairment patients, rigorous clinical trials such as randomized, placebo-controlled trials with neuroimaging techniques are required.

In this study, our main hypothesis would be that brain networks and brain activations focused on frontal regions would be altered according to the disease status and CR level, and that brain connectivity in the frontal regions would change before and after acupuncture stimulation. Next, we aim to investigate the neural substrates of CR and cognitive function using network metrics. Herein, we present a protocol for a randomized, placebo-controlled, parallel-group clinical trial to explore the acute neural changes after acupuncture stimulation according to CR and elucidate the neural substrates of CR according to disease status by applying fNIRS.

## Methods

### Trial Design, Setting, and Aim

This study will be a single-center, block-randomized, placebo-controlled, parallel-group clinical trial. The study will be conducted at the College of Korean Medicine at Sangji University. This protocol complies with the SPIRIT (Standard Protocol Items: Recommendations for Interventional Trials) guidelines [25]. The aim of this study is to investigate the acute neural mechanisms of acupuncture stimulation concerning CR and to identify the neural substrates of CR in the group without cognitive impairment and the group with cognitive impairment using fNIRS.

**Figure 1:**
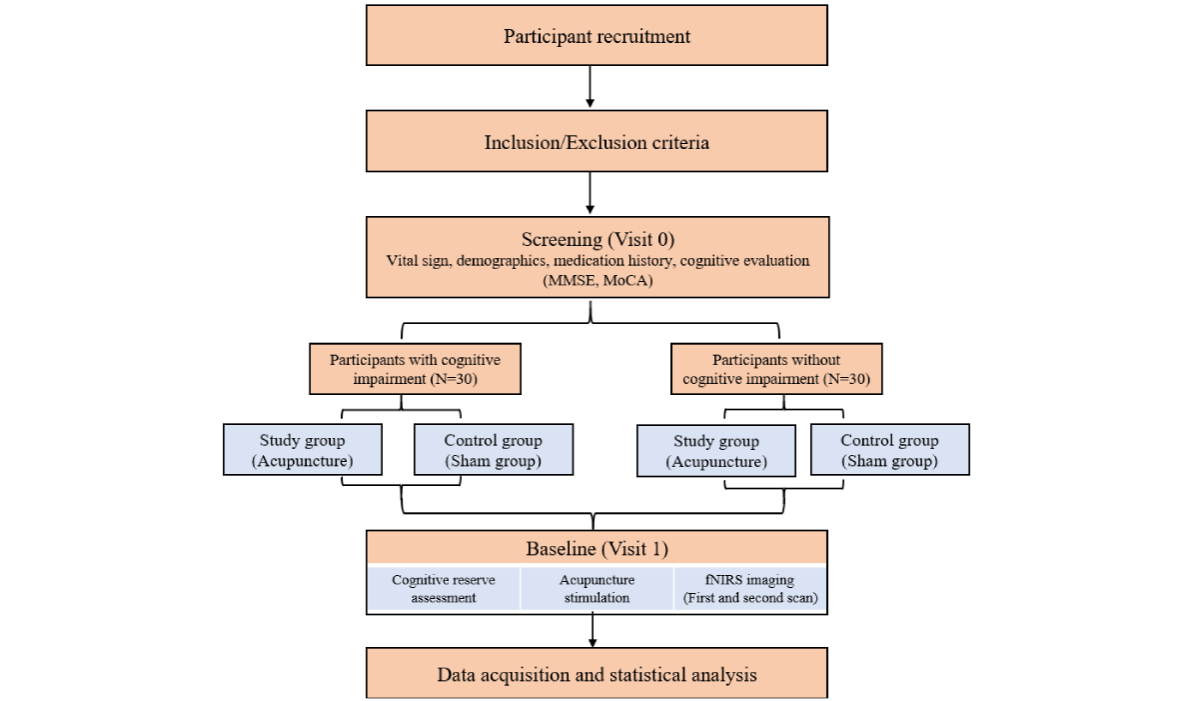
Flowchart of this study. K-MoCA: Montreal Cognitive Assessment (Korean version); MMSE-K: Mini-Mental State Examination (Korean version).

The participants will be divided into 2 equal groups: the verum acupuncture group (study group) and the sham acupuncture group (control group). After each group is confirmed to participate in the study through screening (visit 0), fNIRS scanning and acupuncture manipulation will be conducted at visit 1. Cognitive function assessments, including the Korean version of the Mini-Mental State Examination (MMSE-K) and Montreal Cognitive Assessment (K-MoCA), will be conducted during screening. At visit 1, each participant will be measured twice using fNIRS (first and second scan) before and after acupuncture stimulation. Finally, CR marker assessment, such as the Cognitive Reserve Index Questionnaire (CRIq), will be conducted through interviews in visit 1. The period between visit 0 and visit 1 will not exceed 1 week. Participant recruitment was initiated on August 22, 2023, and the study is scheduled to continue until February 2025. The flow chart is illustrated in Figure 1, and the overall procedure is presented in Table 1.

**Table 1:** Overall procedure of this study.

			Study period		
Time point			Visit 0 (screening)		Visit 1
**Enrollment**					
	Eligibility screen	✓			
	Informed consent	✓			
	Randomization and allocation	✓			
**Intervention**					
	Verum acupuncture group			✓	
	Sham acupuncture group			✓	
**Assessment**					
	Demographics	✓			
	Medical history	✓		✓	
	MMSE-K^a^	✓			
	K-MoCA^b^	✓			
	CRIq^c^			✓	
	fNIRS scan^d^			✓	
**Safety**					
	Vital sign	✓		✓	
	Adverse events			✓	

^a^MMSE-K: Mini-Mental State Examination (Korean version).

^b^K-MoCA: Montreal Cognitive Assessment (Korean version).

^c^CRIq: Cognitive Reserve Index Questionnaire.

^d^fNIRS: functional near-infrared spectroscopy.

### Participants and Recruitment Strategy

#### Eligibility Criteria

The inclusion criteria for healthy controls (the group without cognitive impairment) are as follows: (1) males and females aged ≥19 years without cognitive impairment (MMSE-K≥24, K-MoCA≥23); (2) participants without severe neurological, organic dysfunction; and (3) participants or authorized surrogates who voluntarily sign the informed consent.

The inclusion criteria for the group with cognitive impairment are as follows: (1) males and females aged ≥60 years showing cognitive impairment (MMSE-K<24, K-MoCA<23) or diagnosed with mild cognitive impairment (MCI) or dementia with a clinical dementia rating<3; (2) participants or authorized surrogates who voluntarily sign the informed consent; and (3) participants who can complete this study.

The exclusion criteria for healthy controls (the group without cognitive impairment) are as follows: (1) participants with other brain disorders, alcoholism or drug abuse, neuropsychiatric disease, or taking psychiatric medicines, and severe organic dysfunctions inducing cognitive impairment; and (2) participants with any other reasons that may be considered inappropriate by the researcher.

The exclusion criteria for the group with cognitive impairment are as follows: (1) severe dementia patients with a clinical dementia rating of 3; (2) patients with other brain disorders such as cerebrovascular disease and epilepsy; (3) patients with cognitive impairment induced by another primary disease; (4) patients with severe organic dysfunctions; (5) patients presenting alcoholism or drug abuse, with neuropsychiatric disease or taking psychiatric medicines; and (6) patients with any other reasons that may be considered inappropriate by the researcher.

#### Enrollment, Randomization, and Blinding

Recruitment will be conducted through advertisements on websites and public announcements. Promotion through local communities is also under consideration. The recruitment of participants with cognitive impairment will be promoted through cooperation with community dementia care centers. In this study, in accordance with the recruitment of the participants, the effect of age on brain function will also be investigated; therefore, recruitment in the group without cognitive impairment will not be restricted to older adult participants.

Stratified block randomization will be performed according to sex. Both groups will be assigned in a 1:1 ratio to the study and control groups by applying a block size of 4. Randomization will be conducted using a computer-generated allocation list by an assigned researcher not involved in the assessment or intervention. Participants will be blinded to the type of intervention used. The practitioner will not provide clues regarding allocating information to the participants during the study. Blinding will be maintained until the end of the study. Participants will be asked about their experience of blinding after the end of the study.

#### Sample Size

In this study, the number of participants has been determined statistically. In our hypothesis, the significance level (α) and power (1–β) will be set to 5% and 80%, respectively. Then, an effect size will be calculated using an estimated value of the variability in the MMSE-K and K-MoCA scores based on the literature [26]. As a result, the number of samples came out to be about 10-15 for each group (study or control group) to satisfy the criteria of the normal deviates of significance level and power in the healthy controls and patient groups. The anticipated dropout rate was set to 10%. A similar number of subjects (i.e., 10–15 subjects in each group) can be found in the previous neuroimaging studies [27-29].

#### Study Procedures

All participants will be subjected to measurement of vital signs (blood pressure, pulse rate, and temperature), demographic information, and medical history (past, present, and family). Cognitive function assessments (MMSE-K and K-MoCA) will also be performed during visit 0 (screening). Eligible participants who meet the inclusion and exclusion criteria will receive the schedule for visit 1.

At visit 1, the investigator will check the participant’s vital signs and changes in medication, perform 2 fNIRS scans before and after acupuncture stimulation, and assess the level of CR using CRIq. For medications, the investigator will check for changes but will not specifically allow or restrict certain drugs. According to stratified randomization, the participants will be assigned to 2 acupuncture stimulation groups. Acupuncture stimulation will be performed by a Korean medicine doctor with >3 years of clinical experience. fNIRS scans consist largely of 2 main parts: a 5-minute resting state scan and a task scan of working memory.

#### Cognitive Function Assessment

The investigator will assess 2 cognitive function tests: the MMSE-K and K-MoCA. The MMSE-K is a 30-point questionnaire consisting of 12 questions that are widely used in clinical and research settings to measure cognitive impairment [30]. The K-MoCA is an extensively used 30-point cognitive assessment tool that effectively differentiates MCI [31]. The evaluation of each assessment will be conducted by an investigator blinded to the assigned group.

#### Measurement of Cognitive Reserve

CR assessment will be conducted using the CRIq questionnaire [32]. It includes demographic data and items categorized into 3 sections: education, working activities, and leisure time; each item returns a subscore. It evaluates the CR of an individual by compiling the information associated with the individual’s entire adult life. The quantification of CR will be calculated as the sum of all domains, and the subscore of each domain will also be applied in further correlation analysis. Depending on the mean value of CRIq, it will be dichotomized into the high CR and low CR groups. The investigator will also assess the participants’ bilingualism. The evaluation of each assessment will be conducted by an investigator blinded to the assigned group.

#### fNIRS Imaging

For this experiment, fNIRS equipment (NIRSIT, OBELAB) will be used. fNIRS is a portable near-infrared neuroimaging device that measures hemodynamic variations in cerebral blood by irradiating the cerebral cortex with a near-infrared laser. The equipment irradiates the wavelengths of 780 and 850 nm of laser, which is harmless to humans. It contains 48 channels that can cover the frontal regions of the brain, allowing the quantification of ΔHbO_2_, ΔHbR, and ΔHbT in that area. Based on this, the analysis applied in fMRI, such as graph theory analysis and general linear model analysis, can be used.

**Figure 2:**
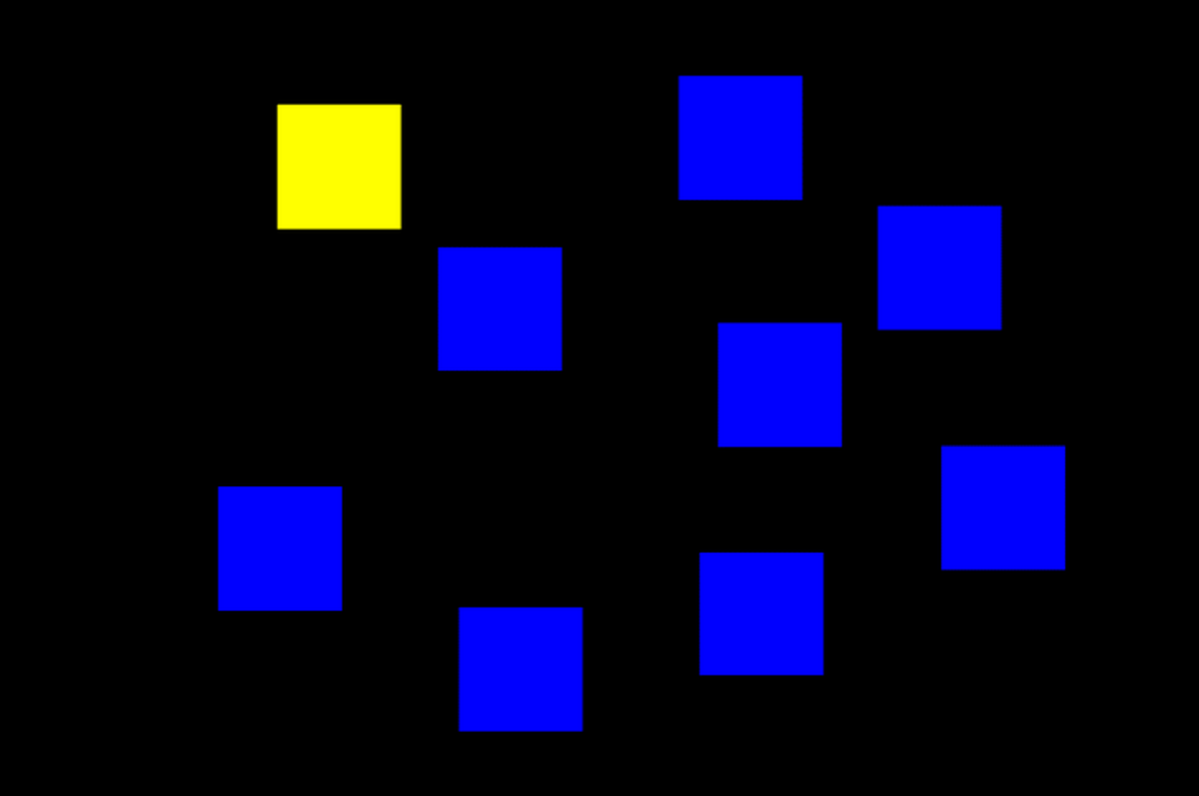
Presentation of Corsi block task.

The fNIRS scans will be conducted before and after acupuncture. The scan consists of 2 parts: a resting state and working memory task scan. The resting-state fNIRS scan lasts for 5 minutes, during which the participant is asked to look at the monitor screen and remain comfortable. The task fNIRS scan is performed while conducting a Corsi block tapping task, a cognitive task related to working memory [33]. This experiment requires participants to tap a spatially separated sequence of up to 9 identical blocks in the same order in which they are presented. The sequences start simple, using 4 blocks, and become more complicated using a maximum of 6 blocks (Figure 2).

### Interventions

In the study group, verum acupuncture stimulation will be conducted for 15 minutes by stainless steel acupuncture (0.25 mm×40 mm, Dongbang acupuncture) on acupoints, which are known to be associated with cognitive functions in previous studies (Baihui [GV20], Sishencong [EX-HN1], Taixi [KI3], Zusanli [ST36], Hegu [LI4], and Taichong [LR3]), and deqi sensation will be induced [24,34,35]. The needle will be lifted and thrust approximately 30-50 mm and twisted and rotated at approximately a 90°-180° angle 60 times/minute (60 Hz). The manipulation will then be performed at 1-minute intervals to maintain the amount of stimulation and achieve deqi sensation. A questionnaire on deqi sensations will be implemented.

For the control group, superficial needling acupuncture (depth of needling: 1-2 mm per nonacupoint) will be performed for 15 minutes using stainless steel acupuncture (0.25 mm×40 mm, Dongbang acupuncture) on nonacupoints. Nonacupoints, which are not related to cognitive function, would be approximately 1 cm away from the acupoints of the study group. The needles for sham acupuncture will remain in place. In the case of superficial needling, manipulation techniques and induction of deqi sensation will not be achieved.

Superficial needling can be a subtype of minimal acupuncture [36]. We thought that the characteristics of acupoints, depth of acupuncture stimulation, intensity of the stimulation, and deqi sensation would all be determinants of acupuncture stimulation. Therefore, we selected minimal acupuncture that lacks these features as a sham control. This type of sham acupuncture has been applied in various studies, including Bell’s palsy, migraine, and chronic musculoskeletal pain syndrome, for a long time [37-40].

### Outcome Measures

This study aims to explore the immediate neural mechanisms of the acute response to acupuncture stimulation associated with CR in normal aging and disease status and identify the neural substrates of CR.

The primary outcomes will be as follows: (1) differences in brain activation and resting-state brain connectivity according to disease status (the groups with vs without cognitive impairment); (2) differences in brain activation before and after acupuncture between study and control groups (verum acupuncture vs sham acupuncture); and (3) differences in brain activation according to CR (high CR group vs low CR group).

Secondary outcomes are brain connectivity or network metrics (graph theoretic metrics) associated with the CRIq index, differences in brain activity between working memory tasks and resting states, and network metrics associated with cognitive function scores (MMSE-K, K-MoCA).

**Figure 3:**
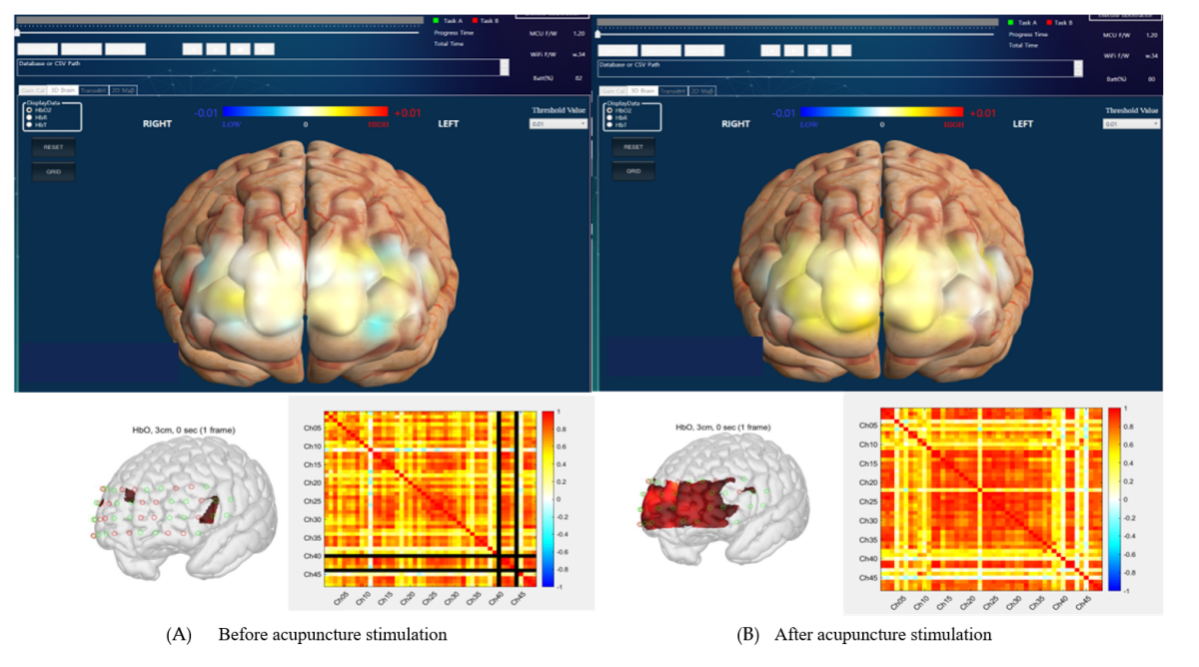
Resting-state brain connectivity matrix before acupuncture stimulation (A) and after acupuncture stimulation (B).

The brain connectivity matrix will be obtained as Pearson correlation between 48 channels using time-series data during the scan period (Figure 3). Network metrics, including the index of efficiency, clustering coefficient, and centrality, can be calculated using the connectivity matrix by the Brain Connectivity toolbox in MATLAB 2023b (The Mathworks).

### Data Collection, Monitoring, and Dropout Criteria

#### Data Collection and Adverse Events Monitoring

Demographic information (age, sex, height, and weight), medical history, and concomitant medication will be collected for each participant at the start of the study. Researchers will record blood pressure, body temperature, respiratory rate, and pulse rate in visits 0 and 1. All adverse events will be fully reported in case report forms, and the association between the adverse events and intervention will be evaluated as not related, possibly related, or related. Severity will be evaluated as mild, moderate, or severe. When severe adverse events, including death, life-threatening events, and the need for hospital admission, occur, the investigator will suspend all or part of the clinical trial and notify other investigators and the Institutional Review Board within 24 hours.

#### Dropout Criteria

The participants will be excluded in the following cases: severe adverse events occur; difficulty proceeding with the trial due to adverse events; the participant or a legal representative wants to stop the trial; the participant withdraws consent to join the trial; the participant fails to finish the interview or the fNIRS scan; and progression of the trial is considered inappropriate by the principal investigator.

### Statistical Analysis

Only data from participants who completed the fNIRS scan, cognitive function test, and measurement of CR will be contained in the statistical analysis. All categorical data are presented as frequencies or percentiles and will be analyzed using the chi-square or Fisher’s exact test. All numerical data will be presented as the mean and standard deviation and analyzed using an independent *t* test or Wilcoxon rank-sum test. Preprocessing and analyses of fNIRS data will be performed using MATLAB and the NIRSIT analysis tool.

A two-sample *t* test will be performed to compare the brain activations and connectivity between the groups with and without cognitive impairment. A paired *t* test will be conducted to compare the brain activations and connectivity before and after acupuncture stimulation. The CR values from CRIq will be applied for correlation analysis in the form of continuous variables or divided into binary groups (high and low CR) by mean values for subgroup analysis. Since the expression of CR may appear differently depending on the disease status, a subgroup analysis will be conducted by dividing the healthy control and the patient group. Mixed model analysis will be adjusted to determine the interaction effect of intervention and CR in both groups. Pearson correlation will determine the CR-related brain connectivity or clinical score–related metrics. If there is a significant age difference between 2 groups, the age factor will be added as a covariate in the analysis to adjust the effect of age as much as possible.

Statistical significance in fNIRS imaging would be explored using uncorrected and corrected levels (false discovery rate and family-wise error). At the uncorrected level, uncorrected *P*<.001 will be used, and at the corrected level, *P*<.05 will be applied.

### Data Management

The investigators will collect and record the medical information in each participant’s case report form. All study data, including all patient confirmations, all original signed informed consent forms, and detailed original records, will be retained by the study institution for 3 years after the end of the trial.

### Quality Control

Important protocol modifications must be reported to the IRB. The approval period of the trial is 1 year, and after 1 year, regular reports must be submitted to the IRB, and re-approval should be obtained. Before the beginning of the trial, all investigators involved in the intervention will be trained in the process of the trial. In total, 2 independent research assistants will check the data and case report forms to avoid mistyping or errors during the trial. There is no plan for early termination of the study before February 2025. If the target number of participants is complete, additional recruitment may proceed depending on the research funding status.

### Ethical Considerations

The trial will be performed in accordance with the Declaration of Helsinki. This trial has been approved by the Institutional Review Board of Sangji University (1040782- 230426-HR-04-112). The protocol has been registered with the Clinical Research Information Service of the Republic of Korea (number KCT0008719), one of the WHO ICTRP Primary Registries. Written informed consent will be obtained from each participant or their legal representative. All data will be anonymized, and the identification information of all documents will be recorded according to the identification code. The information will be kept confidential in a locked place. Participants in this study will be paid a small compensation fee, approximately 50,000 won (equivalent to approximately US $30), for participation in the study.

## Results

The recruitment and enrollment of the study began in August 2023, and to date, there have been 50 participants, divided into 20 in the group with cognitive impairment and 30 in the unimpaired group. Within the group with cognitive impairment, 16 participants (80%) had dementia and 4 subjects had MCI. The percentages of female participants in the group with cognitive impairment and the group without cognitive impairment were 70% and 57%, respectively. Ongoing recruitment is in progress. The protocol version used in this study is V.1.0 (2023.06.12). This study will continue until February 2025, after which the communication of final results will be made in accordance with the CONSORT checklist.

## Discussion

### Overview

Dementia affects an estimated 57 million people worldwide, and AD is the most common dementia-inducing disease [41]. The neuropathologic hallmark of AD is extracellular amyloid beta deposition and neurofibrillary tangles consisting of phosphorylated tau protein [42]. Although various therapeutic drugs are being developed with the advancement of medical technology, there is no definitive treatment for AD. Therefore, early diagnosis and prevention are important for cognitive impairment disorder.

At first, CR originated from the observations of the mismatch between the pathological degree of brain damage and clinical symptoms. In a postmortem study, there were neuropathological alterations comparable to dementia; however, the participants had no symptoms of dementia in their lives [43]. In this context, CR is considered a factor mediating the relationship between the neuropathology of the disease and clinical manifestation and has clinical importance as a prognostic factor of the disease trajectory [44]. There are several CR surrogate markers, including years of education, occupation, leisure activities, composite scores, questionnaires, and residual methods using imaging markers. This study applies a CRIq, which consists of subscores of lifelong education, occupation, and leisure activity as the main CR proxy.

Acupuncture has been used to treat several neurological diseases in East Asian countries. Several studies have revealed the effects and mechanisms of acupuncture in MCI or AD using neuroimaging methods. Moreover, studies applying fNIRS have reported the neural mechanisms of acupuncture manipulation in patients with MCI and healthy participants [45,46]. However, there have been no studies on the neural mechanisms of acupuncture stimulation in relation to CR.

This randomized, placebo-controlled clinical trial aims to investigate the neural substrates of CR in both the normal group and the group with cognitive impairment and explore the neural mechanisms of acupuncture stimulation in relation to CR using fNIRS. The potential significance of this research lies in elucidating the connection between acupuncture and CR, thus providing insights into the therapeutic possibilities for cognitive impairments. We expect to contribute to identifying neural mechanisms of acupuncture stimulation in cognitive impairment in relation to CR. This study could provide a basis for further study about the long-term effects and mechanisms of acupuncture treatment in cognitive impairment.

### Innovations and Limitations

This is the first study to identify the association between CR, an important factor related to the prognosis of dementia, and acupuncture stimulation. The application of the neuroimaging method (fNIRS) can provide new insights into the neural mechanisms of complementary medicine in the field of convergence research. This randomized, controlled trial can contribute to elucidating the genuine neural response to the acupuncture stimulation. However, as a cross-sectional study, it is not possible to identify the long-term effect of acupuncture treatment on cognitive impairments and explore the longitudinal neural mechanism of acupuncture treatment. Additionally, to ensure the reliability of the data, we still further expand the sample size.

### Conclusions

This study will provide data on the neural substrates of acupuncture stimulation associated with CR as well as brain network alterations depending on disease status. More importantly, if the acute neural mechanisms of acupuncture stimulation are identified, the results could serve as a basis for the long-term effects of acupuncture treatment on cognitive impairments. This study could provide a scientific foundation for further clinical application of acupuncture as a complementary medicine for cognitive impairments, which induce a high socioeconomic burden.

## Abbreviations

AD: Alzheimer disease
CR: cognitive reserve
CRIq: Cognitive Reserve Index Questionnaire
fMRI: functional magnetic resonance imaging
fNIRS: functional near-infrared spectroscopy
HbO2: oxyhemoglobin
HbR: deoxyhemoglobin
HbT: total hemoglobin
MCI: mild cognitive impairment
MMSE: Mini Mental Status Examination
MoCA: Montreal Cognitive Assessment
SPIRIT: Standard Protocol Items: Recommendations for Interventional Trials

## Data Availability

All study data will be retained by the study institution for 3 years after the end of the trial. All investigators participating in this trial will have access to the final dataset after recruitment is completed. The datasets generated or analyzed during this study are available from the corresponding author on reasonable request.
